# Meningeal lymphatic-associated brain swelling in acute stroke

**DOI:** 10.1371/journal.pone.0342643

**Published:** 2026-02-12

**Authors:** Rohan Mahesh Patil, Dong Bin Back, Gen Hamanaka, Rakhi Desai, Ayumi Hayakawa, Su Jing Chan, Bum Ju Ahn, Giuseppe Pignataro, Kazuhide Hayakawa, Elga Esposito

**Affiliations:** 1 Neuroprotection Research Laboratory, Departments of Radiology and Neurology, Massachusetts General Hospital and Harvard Medical School, Charlestown, Massachusetts, United States of America; 2 Division of Pharmacology, Department of Neuroscience, School of Medicine, University “Federico II”, Naples, Italy; School of Medicine, Tokyo Women's Medical University, JAPAN

## Abstract

Brain meninges contain lymphatic vessels that play roles in clearance of extracellular solute in the central nervous system. But, whether and how the system is involved in acute stroke remains to be fully explored. Here, we show the VEGF-C-Flt4 pathway involvement in brain swelling in acute phase of ischemic stroke in rats. We first confirmed that a prototypical lymphatic mediator VEGF-C was upregulated in brain endothelium and secreted into CSF. Concomitantly, VEGF-C receptor Flt4 was increased in the meninges but not in peri-infarct cortex. Next, we isolated lymphatic endothelial cells from rat meninges using LYVE-1 antibody-conjugated magnetic beads. An *in vitro* standard matrigel assay confirmed that isolated LYVE1 + cells increased ring-like structures by treatment with VEGF-C or conditioned media from injured brain endothelium subjected to oxygen-glucose deprivation, whereas immunodepletion of VEGF-C from endothelial media decreased the effect. Finally, blocking Flt4 tyrosine kinase *in vivo* suppressed the acute increase of lymphatic endothelial cells accompanied by reduction of brain swelling. Collectively, the proof-of-concept study suggests that the VEGF-C–Flt4 signaling pathway contributes to brain swelling during the acute phase of ischemic stroke by activating meningeal lymphatic endothelial cells. Targeting this pathway may offer a new approach to mitigate stroke-induced inflammation and edema.

## 1. Introduction

While the brain has been considered to lack lymphatic vessels, lymphatic-like clearance system has long been found in the central nervous system (CNS) [1–4]. More recently, the detailed structure of lymphatic vessels were identified in adult mouse meninges, and the brain meningeal lymphatic system appeared to be responsible for controlling immune cells to entry and exit CNS as well as clearing excess interstitial fluid from the CNS [5–7]. Importantly, MRI studies have revealed the presence of lymphatic vessels in the human meninges, which drain cerebrospinal fluid (CSF) from the brain into cervical lymph nodes [8,9]. Collectively, these findings establish the meningeal lymphatic system as a clinically relevant component of CNS homeostasis and a potential contributor to neurological disease. Although MLVs are anatomically located outside the brain parenchyma, they function as a critical downstream drainage pathway for brain-derived fluids. Interstitial fluid and solutes exit the parenchyma along perivascular routes described by the glymphatic system and enter the subarachnoid space, where they are subsequently cleared through meningeal lymphatic vessels. Through this functional coupling, MLVs play an essential role in regulating brain fluid balance despite their extra-parenchymal location [10].

It has been discussed that meningeal lymphatics and CSF drainage play a critical role in the formation of cerebral edema in ischemic stroke [11–13]. Under normal conditions, meningeal lymphatic vessels help maintain fluid homeostasis in the central nervous system by facilitating the clearance of interstitial fluid and extracellular solutes [14]. However, during ischemic stroke, this drainage pathway may become impaired due to inflammation, vascular damage, or altered signaling mechanisms [15]. How signals originating from the injured brain influence meningeal lymphatic endothelial function, and how this interaction contributes to impaired drainage and edema formation, remains poorly understood.

Vascular endothelial growth factor-C (VEGF-C) and its receptor Flt4 (VEGFR-3) are central regulators of lymphatic vessel maintenance, permeability, and function [16–18]. We hypothesized that dysregulation of VEGF-C–Flt4 signaling in meningeal lymphatic endothelial cells contributes to impaired lymphatic drainage after stroke, thereby exacerbating brain swelling. In this proof-of-concept study, we combined in vitro lymphatic endothelial cell approaches with in vivo stroke models to examine meningeal lymphatic endothelial responses to ischemic injury. Furthermore, we tested whether modulation of meningeal lymphatic VEGF-C–Flt4 signaling could attenuate post-stroke brain edema. By defining the role of this pathway in meningeal lymphatic dysfunction, this study aims to clarify the mechanistic link between lymphatic signaling, brain fluid drainage, and edema formation after stroke.

## 2. Materials and methods

### 2.1. Animals

All experiments (n = 54) were performed following an institutionally approved protocol in accordance with National Institutes of Health guidelines and with the United States Public Health Service's Policy on Human Care and Use of Laboratory Animals and following Animals in Research: Reporting *In vivo* Experiments (ARRIVE) guidelines (ethics approval reference number: 2012N000165). Male Sprague-Dawley rats (320–340 g) were housed in pathogen-free facilities with 12h day and night cycles. All animals were randomly allocated to treatment groups, and final outcomes for FACS, infarction, and brain swelling were evaluated in a blind manner. 3 days before surgery animals were handled for 10 minutes each for 3 days.

### 2.2. Focal ischemia

All animals were anesthetized with isoflurane (1.5%) in 30%/70% oxygen/nitrous oxide. Transient focal ischemia was induced introducing a 5-O surgical monofilament nylon suture (Doccol) from the external carotid artery into the internal carotid artery and advancing it to the branching point of the MCA. Cerebral blood flow was monitored, by continuous laser doppler flowmetry (LDF) (Perimed, North Royalton, OH, U.S.A.), in the area of the right MCA to confirm adequate occlusion. Animals that did not have a significant reduction to less than 30% baseline LDF values during MCAO were excluded and euthanized (n = 3). After occluding the MCA for 100 minutes, the monofilament suture was gently withdrawn in order to restore blood flow, and LDF values were recorded for 10 minutes after reperfusion. Rectal temperature was monitored and maintained at 37°C ± 0.5°C with a thermostatically controlled heating pad during surgery and with a heating lamp for 4 hr after surgery. At 24hr after stroke onset, the animals were anesthetized with isoflurane (1.5%) in 30%/70% oxygen/nitrous oxide and underwent transcardial perfusion with phosphate-buffered saline (PBS). Brains were extracted, placed within a brain matrix and cut in 8 coronal sections of 1 mm each. The sections were then incubated in 2% TTC in 1 x phosphate buffered saline (PBS) for 10 min at 37°C. Infarction volumes were quantified using the “indirect” morphometric method with Image J software. Brain swelling was calculated by the percentage of ipsilateral hemisphere to contralateral hemisphere. CSF was collected, while animals were anesthetized with isoflurane (1.5%) in 30%/70% oxygen/nitrous oxide, from the rat cisterna magna at 3 hr and 24 hr after ischemia. The animals were anesthetized and positioned in a stereotaxic frame, the rat head was flexed downward at approximately 45 degrees. A capillary tube was used to penetrate into the cisterna magna through the dura mater. The CSF flowed into the capillary tube and collected into a tube.

Ethiqa XR (Fidelis Animal Health, Inc), a substance consisting of a buprenorphine extended-release injectable suspension, was injected 3.25 mg/kg subcutaneusly 30 mins before MCAO. After surgery, all animals were closely monitored, every 12 hours, and rats that showed unacceptable signs were euthanized (n = 5). Signs of unacceptable stress include inability of eat and drink, ruffled fur/inadequate grooming, excessive lack of spontaneous activity, excessive weight loss of more than 15% compared to normal age-matched animals (weighted every day). If rats were unable to bear weight on one side or no spontaneous movement or barrel rolling, animals were euthanize immediately.

### 2.3. Primary rat meningeal lymphatic endothelial culture

At 24h after stroke the animals were euthanized by isoflurane anesthetic overdose (5% isoflurane followed by decapitation) and primary meningeal lymphatic endothelial cultures were prepared from meninges of postnatal d1 to d2 Sprague-Dawley rat embryos. Briefly, meninges were dissected, then cell suspensions were prepared. Biotin-conjugated LYVE-1 antibody (10 μg, NB100-725B, NOVUS biologicals) was mixed with CELLection Dynabeads (11533D, Thermo Fisher Scientific) coated with recombinant streptavidin (100 µl, 4x10^7^ beads) for 30 min. After removing free floating antibody, beads were mixed with cell suspensions for 20 min at 4^o^C. Following removal of beads from cells with DNase solution, cells were plated and cultured with EBM2 plus supplement growth factors for 5−7 days. Cells were then stained for LYVE-1 (13-0443-80, eBioscience) and CD31 (102423, BioLegend) antibodies, the immunostaining confirmed that the isolated cells were indeed lymphatic endothelial cells (Supplementary 1).

### 2.4. Oxygen-glucose deprivation (OGD) and reoxygenation

OGD experiments were performed using a specialized, humidified chamber (Heidolph, incubator 1000, Brinkmann Instruments, Westbury, NY) kept at 37^o^C, which contained an anaerobic gas mixture (90% N_2_, 5% H_2_, and 5% CO_2_). To initiate OGD, culture medium was replaced with deoxygenated, glucose-free Dulbecco's modified Eagle medium (Life Technology, 11966−025). After 3 h challenge, rat brain endothelial cells were removed from the anaerobic chamber, and the OGD solution in the cultures was replaced with maintenance medium. Cells were then allowed to recover for 18 h in a regular incubator.

### 2.5. *In vitro* tube formation assay

The standard Matrigel assay was used to assess the spontaneous formation of capillary-like structures by lymphatic endothelial cells. μ-slide angiogenesis chamber (81506, Ibidi) were coated with 11 μL of cold Matrigel and allowed to solidify at 37˚C for 20 minutes. Cells (1.2 × 10^4^ cells/well) were seeded in those plates, and incubated at 37˚C for 18 hr with or without VEGF-C (1, 10, 100 ng/mL) or VEGF-A (1, 10, 100 ng/mL) treatment.

### 2.6. FACS analysis

FACS analysis was performed as described before [19,20]. Tissues collected from meninges and peri-infarct cortex are gently minced and then digested at 37°C for 30 min with an enzyme cocktail. FACS analysis was performed using a no labeled control for determining appropriate gates, voltages, and compensations required in multivariate flow cytometry.

### 2.7. Western blot analysis

Western blot was performed as described before [19,20]. Protein samples were prepared by Pro-PREPTM Protein Extraction Solution (INB17081, BOCA SCIENTIFIC). Each sample was loaded onto 4–20% Tris-glycine gels. After electorophoresis and transferring to nitrocellulose membranes, the membranes were blocked in Tris-buffered saline containing 0.1% Tween 20 and 0.2% I-block (T2015, Tropix) for 90 min at room temperature. Membranes were then incubated overnight at 4°C with following primary antibodies, anti-β-actin (1:1,000, A5441, Sigma-aldrich), anti-VEGF-C antibody (1:200, sc-374628, Santa Cruz), anti-VEGF-A antibody (1:200, sc-507, Santa Cruz). After incubation with peroxidase-conjugated secondary antibodies, visualization was enhanced by chemiluminescence (GE Healthcare, NA931- anti-mouse, or NA934- anti-rabbit, or NA935- anti-rat). Optical density was assessed using the NIH Image analysis software.

### 2.8. Immunoprecipitation

Brain endothelial conditioned media were collected after 3h OGD/18h reoxygenation. According to the manufacture's instruction, anti-VEGF-C antibody (20 μg, sc-374628, Santa Cruz) or anti-VEGF-A antibody (20 μg, sc-507, Santa Cruz) and Dynabeads Protein G Immunoprecipitation kit (10007D, Life technologies) were used to deplete VEGFs from media. Following immunoprecipitation, the depletion of VEGF-C or VEGF-A was assessed by western blot.

### 2.9. Intracerebroventricular and intra-striatum administration

Male Sprague Dawley (SD) rats were positioned on a stereotaxic frame and a 23-g stainless steel guide cannula was implanted into the right lateral ventricle using the stereotaxic coordinates from the bregma: −0.9 mm caudal, 1.4 mm lateral and 3.8 mm from the skull for i.c.v. injection and −0.2 mm caudal, 3.0 mm lateral and 6.0 mm for intra-striatum injection. The same procedure was used to inject (i.c.v.) MAZ51 (50 ng/5 μL) [21] or respective vehicle (PBS) immediately after the stroke onset in a randomized fashion.

### 2.10. Statistical analysis

Results were expressed as mean ± SEM. Sample sizes were estimated from pilot data and the literature to achieve the planned statistical power. When only two groups were compared, unpaired t-test was used. Multiple comparisons were evaluated by one-way ANOVA followed by Tukey test. *P* < 0.05 was considered to be statistically significant.

## 3. Results

### 3.1. VEGF-C is upregulated in brain endothelium and increased in CSF after focal ischemia in male Sprague Dawley (SD) rats

**Fig 1:**
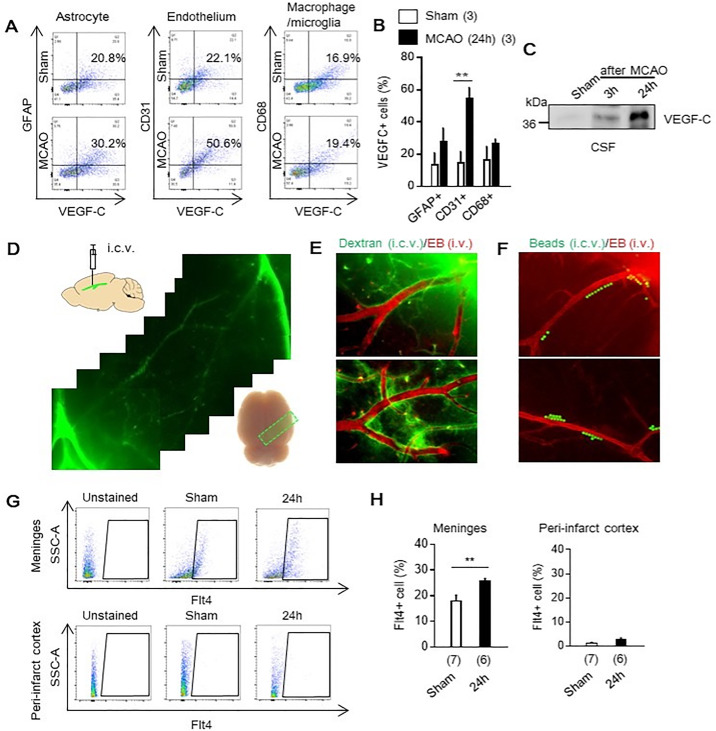
Acute increase of lymphatic endothelial cells via VEGFR3 (Flt4) after transient focal cerebral ischemia in Sprague Dawley (SD) rats: Male SD rats were subjected to 100 min of transient focal cerebral ischemia. **A, B.** Flow cytometry analysis showed that VEGF-C appeared to be increased in CD31 + endothelial cells without significant changes appearing in GFAP+ astrocytes and CD68 + microglia/macrophage (n = 3). **P < 0.01. **C.** Western blot showed release of VEGF-C into CSF after focal ischemia in SD rats. **D-F.** Intracerebroventricular (i.c.v.) infusion of dextran-FITC (40 kDa) or 15 μm micro-beads-FITC together with intravenous (i.v.) injection of Evans blue in brains of normal rats confirmed CSF drainage pathway via perivascular space. **G, H.** Male SD rats were subjected to 100 min of transient focal cerebral ischemia, then meningeal area, followed by isolating peri-infarct cortex, was isolated. Flow cytometry analysis demonstrated that Flt4 positive lymphatic endothelium were significantly increased in meninges at 24 hr after stroke, but they were barely changed in peri-infarct cortex. (n = 4-7). **P < 0.01. All values are mean + /- SEM.

VEGF-C is known as a prototypical mediator for lymphatic activation and lymphangiogenesis [16,22]. However, lymphatic endothelial cells do not express VEGF-C but instead express its receptor VEGFR3 (Flt4). We therefore investigated whether VEGF-C is produced by brain-associated cells and released after stroke to signal to meningeal lymphatic vessels. Male SD rats were subjected to 100 min of transient focal cerebral ischemia. Peri-infarct cortex was isolated at day 1 post-ischemia and processed for flow cytometry. VEGF-C expression was increased predominantly in CD31 ⁺ blood endothelial cells, with smaller changes observed in GFAP⁺ astrocytes and CD68 ⁺ microglia/macrophages (Fig 1A, 1B). Importantly, western blot analysis demonstrated increased levels of VEGF-C in the CSF within 24 hours after focal ischemia (Fig 1C), supporting the idea that brain- or vascular-derived VEGF-C is released into the CSF and subsequently reaches VEGFR3-expressing meningeal lymphatic vessels to promote their activation.

### 3.2. Lymphatic endothelial marker VEGFR-3 (Flt4) was increased in the meninges after focal ischemia

CSF is produced in the lateral ventricles and flows through the ventricular system into the subarachnoid space, where it exchanges with interstitial fluid along perivascular (glymphatic) pathways and ultimately drains via meningeal lymphatic vessels [4]. To reconfirm this established CSF drainage pathway, we performed intracerebroventricular (i.c.v.) infusion of dextran-FITC (40 kDa) or 15 μm FITC-labeled microbeads, together with intravenous (i.v.) injection of Evans blue, in normal rats (Fig 1D–1F). These tracers followed the canonical CSF circulation and drainage route toward the meninges, supporting the concept that CSF-borne components can reach and influence meningeal lymphatic vessels. We then collected meningeal tissue and peri-infarct cortex to assess lymphatic activation after focal ischemia. Flow cytometry analysis showed a significant increase in Flt4 ⁺ lymphatic endothelial cells in the meninges at 24 h after stroke, whereas Flt4 expression was minimal and unchanged in the peri-infarct cortex (Fig 1G, 1H).

### 3.3. VEGF-C and VEGF-A increased angiogenic response in meningeal lymphatic endothelial cells in vitro

**Fig 2:**
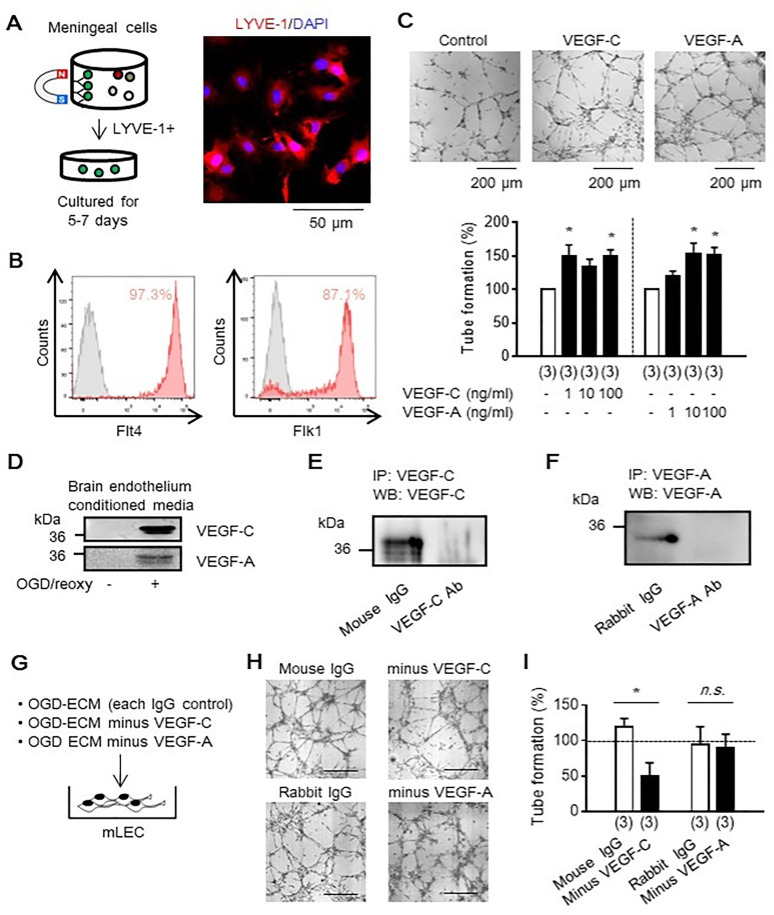
Meningeal lymphatic endothelial angiogenesis through VEGF signaling *in vitro.* **A.** LYVE-1 antibody-conjugated magnetic beads were used for lymphatic endothelial isolation. Scale: 50 μm. **B.** FACS analysis demonstrated that the isolated LYVE-1 positive cells highly expressed a lymphatic endothelium marker Flt4 (VEGF-C receptor), and VEGF-A receptor, Flk1. **C.** In vitro tube formation assay revealed that VEGF-C effectively induced lymphatic endothelial angiogenesis in vitro compared to VEGF-A (n = 3). Scale: 200 μm. **D.** Western blot showed that brain endothelium (RBE.4.) produced VEGF-C and VEGF-A into the cultured media following oxygen-glucose deprivation (OGD). **E, F.** Western blot analysis confirmed VEGF depletion from brain endothelial conditioned media following immunoprecipitation for each target (VEGF-C or VEGF-A). **G.** Schematic for media transfer experiment. Brain endothelium-derived conditioned media were transferred into meningeal lymphatic endothelial cells to assess lymphangiogenesis. **H, I.** After 24 hours, VEGF-C depletion significantly reduced the ability of endothelial conditioned media to promote angiogenic response in meningeal lymphatic endothelial cells, but VEGF-A depletion did not influence lymphangiogenesis compared to control IgG (n = 3). Scale: 200μm. *P < 0.05. All values are mean + /- SEM.

Here we investigate whether lymphatic endothelial cells could be isolated and cultured for functional analysis. Primary rat meningeal lymphatic endothelial cells were isolated by LYVE-1-conjugated magnetic beads, then cultured for 5–7 days (Fig 2A). Flow cytometry analysis confirmed that the isolated cells expressed Flt4, which is primarily expressed in lymphatic endothelial cells, and VEGF-A receptor Flk1, mostly expressed in endothelial cells (Fig 2B). An in vitro matrigel assay demonstrated that VEGF-C effectively increased ring formation in meningeal lymphatic endothelium compared to VEGF-A (Fig 2C).

### 3.4. Brain endothelium-derived VEGF-C increased meningeal lymphangiogenesis in vitro

An important question at this point is whether VEGF-C produced by brain cells after stroke promotes lymphangiogenesis. To address this question, we asked whether stressed brain endothelium could actively produce VEGF-C after oxygen-glucose deprivation (OGD) *in vitro*. We could confirm that brain endothelial cells produced VEGF-C and VEGF-A into the media (Fig 2D). Next, we designed media transfer experiment with or without VEGF depletion. To deplete VEGF-C or VEGF-A, immunoprecipitation (IP) was conducted. Western blot following IP demonstrated that each VEGF depletion was successfully performed (Fig 2E, 2F). The depletion of VEGF-C significantly reduced the number of rings in the isolated lymphatic endothelium, but depleting VEGF-A was not sufficient to decrease the ring formation (Fig 2G-2I).

### 3.5. Blocking Flt4 tyrosine kinase attenuated meningeal lymphatic activation and brain swelling after focal ischemia in rats

**Fig 3:**
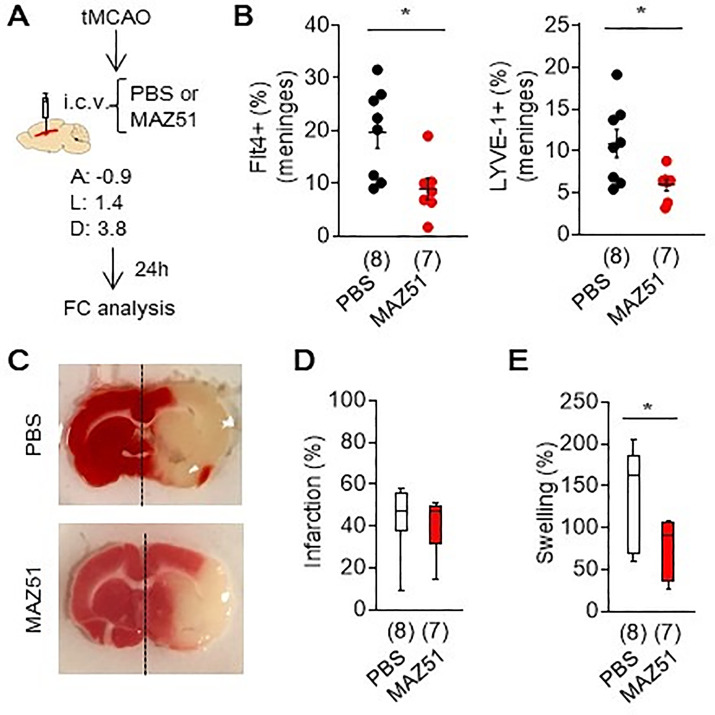
An inhibition of flt4 tyrosine kinase with MAZ51 decreased brain swelling after focal cerebral ischemia. **A.** Flt4 tyrosine kinase inhibitor, MAZ51 (50 ng/5 µL) was intraventricularly injected immediately after 100 min of transient focal ischemia, then meninges in ipsilateral hemisphere were isolated at 24 hr after stroke onset for FACS analysis. **B.** Lymphatic endothelial markers, Flt4 and LYVE-1 positive cells were significantly decreased by MAZ51 treatment (n = 7-8). *P < 0.05, values are mean + /- SEM. **C-E** TTC staining showed no significant change in infarct size. Box-and-whisker plots in **D-E** show reduced brain swelling in the ipsilateral hemisphere with MAZ51 (n = 7–8, boxes = 25th–75th percentiles, line = median, whiskers = min–max, P < 0.05).

Male SD rats were subjected to 100 min transient focal cerebral ischemia. To assess VEGF-C-Flt4 signaling involved, Flt4 tyrosine kinase inhibitor MAZ51 was injected through lateral ventricles (Fig 3A). Blocking Flt4 tyrosine kinase signaling with MAZ51 decreased Flt4 and LYVE-1 positive lymphatic endothelial cells in meninges at 24 hr after focal ischemia (Fig 3B). TTC staining demonstrated that 100 min of focal ischemia produced large infarction (Fig 3C), and MAZ51 did not significantly decreased the infarction at 24 hours post-stroke (Fig 3D). However, brain swelling was decresed in the acute phase of ischemic stroke (Fig 3E).

## 4. Discussion

Over the last two decades, many progresses have been made in the stroke research. However, clinical trials continue to fail, thus stressing the need of new different approaches. More recently, emerging evidences suggest that CNS lymphatic vessels may be involved in clearing excess interstitial fluid [5,6]. Despite the discovery of the presence of lymphatic vasculature, the signals and mechanisms of CNS lymphatics in stroke remain to be fully elucidated. In the present study, we found lymphatic involvement in stroke showing that (i) functional lymphatic endothelial cells were present in rat meninges, (ii) meningeal lymphatic endothelial cells were increased after transient focal ischemia, and (iii) blockade of Flt4 tyrosine kinase suppressed acute increase of lymphatic endothelium along with reducing brain swelling. Taken together, these findings may provide a new therapeutic strategy to suppress cerebral edema caused by meningeal lymphatics in acute stroke.

VEGF was discovered for the first time in 1989 and it is considered a major mediator of both vasculogenesis and angiogenesis [23]. Several isoforms of VEGF exist due to alternative splicing of a single gene that forms polypeptides of different sizes. These isoforms are thought to have distinct but overlapping functions in angiogenesis [23]. VEGF has been implicated in brain ischemia, where it can mediate angiogenesis and vascular permeability [24]. Although the majority of studies tend to focus on VEGF-A, it has been reported that VEGF-C is also upregulated in peri-infarct areas [25]. It is known that VEGF-C can bind VEGFR-2 (Flk1) in blood vascular endothelial cells, and VEGFR-3 (Flt4) in lymphatic endothelial cells. Additionally, VEGF-C is involved in lymphangiogenesis and inflammation induces lymphangiogenesis by up-regulation of VEGFR-3 [16,22,26]. Recent studies have highlighted the role of meningeal lymphatics in influencing stroke outcomes, particularly through VEGF-C signaling  [27]. Our initial study suggested that VEGF-C/VEGFR3 signaling may contribute to post-stroke inflammation [21]. However, later work showed that AAV-VEGF-C delivery into the CSF protects against stroke by expanding meningeal lymphatics and enhancing CSF drainage [28]. Subsequent studies further revealed a damaging role for VEGF-C in the acute phase and a pro-angiogenic effect in the chronic phase [29].

In line with this, another study showed that blocking VEGF-C via soluble VEGFR-3 overexpression reduced edema, inflammation, and vascular leakage  [30]. However, a more recent study reported that VEGF-C administration improved stroke-induced neurological deficits [31], suggesting that the role of VEGF-C in stroke is complex and context-dependent. In our study, we found that pharmacological inhibition of meningeal lymphatic Flt4 signaling with MAZ51 significantly reduced brain swelling, indicating that meningeal lymphatic vessels may represent a promising therapeutic target for modulating the inflammatory response during the acute phase of ischemic stroke. Importantly, while MAZ51 was administered intracerebroventricularly in the present study for mechanistic purposes, we have previously demonstrated that MAZ51 can be delivered intranasally and effectively reach the brain after stroke [21]. This noninvasive route provides a more clinically feasible approach for targeting VEGFR3 signaling in meningeal lymphatics, highlighting the translational potential of our findings.

Nevertheless, there are a few caveats that need to be addressed in future studies. First, we focused on VEGF-C - Flt4 signaling to assess the involvement of CNS lymphatics in stroke pathophysiology because it is a key factor for lymphatic inflammation and its receptor Flt4 is enriched in lymphatic endothelium [26,32]. However, many factors including NRP2, BMP9, and Eph-Ephrin [33–35] may also activate lymphatic system. How VEGF-C interacts with other signaling pathways in stroke remains to be fully dissected. Second, although blocking meningeal lymphatic activation in acute stroke reduced brain swelling in our study, this effect is context-dependent. Enhanced MLV function can be protective in other conditions, such as improving drainage in brain cancer, facilitating repair after traumatic brain injury, or promoting recovery from subarachnoid and subdural hemorrhage [36–39]. These apparent contradictions highlight the biphasic and context-specific actions of CNS lymphatics, where timing, disease type, and lymphatic modulation critically influence outcomes. Third, some experiments, including FACS analyses and tube formation assays, were performed with small sample sizes (n = 3), which may limit statistical power and generalizability. Forth, while inhibition of Flt4 signaling significantly reduced acute brain swelling, this study did not directly assess functional neurological outcomes. Brain edema is a key contributor to secondary injury after ischemic stroke, but reduced swelling does not necessarily translate into improved neurological function. Future studies will therefore be required to determine whether modulation of meningeal lymphatic Flt4 signaling improves behavioral and long-term functional recovery after stroke, including sensorimotor and cognitive outcomes. Finally, we showed that blocking lymphatic activation in meninges in acute stroke decreased brain swelling, however how this might affect inflammatory response in the brain needs to be investigated. It is known that lymphatic inflammation and lymphangiogenesis have biphasic actions. It can worsen damage [40] but can also help resolve injury and promote repair [41]. Further studies are required to investigate how lymphatic system in the CNS influences stroke recovery over time.

## 5. Conclusion

It has been proposed that the brain may possess a lymphatic-like system [2,42]. But, whether or how lymphatic system can participate in stroke pathophysiology remains to be fully explored. Here, we show that meningeal lymphatic vessels may regulate post-stroke cerebral edema. Meningeal lymphatic vessels were activated via VEGF-C-Flt4 signaling while treatment with MAZ51 reduced brain swelling in acute phase after stroke. Further investigation into these mechanisms may lead to novel therapeutic opportunities targeting CNS lymphatics after stroke.

## Supporting information

S1 File Validation of Meningeal Lymphatic Endothelial Cell Identity. **A.** Immunohistochemistry showing double staining for LYVE-1 (marker of lymphatic endothelial cells) and CD31 (marker of endothelial cells) in isolated meningeal cell preparations. The majority of LYVE-1 ⁺ cells co-express CD31, indicating that the isolated LYVE-1 ⁺ population is predominantly composed of meningeal lymphatic endothelial cells. Representative images are shown.
(PDF)

S1 Fig Fig 1C. (PNG)
