# Supplementary figures and images for: Meningeal lymphatic-associated brain swelling in acute stroke
Source: PLoS One. 2026 Feb 12;21(2):e0342643. doi: 10.1371/journal.pone.0342643 (PMC12900329; doi:10.1371/journal.pone.0342643)

**A**

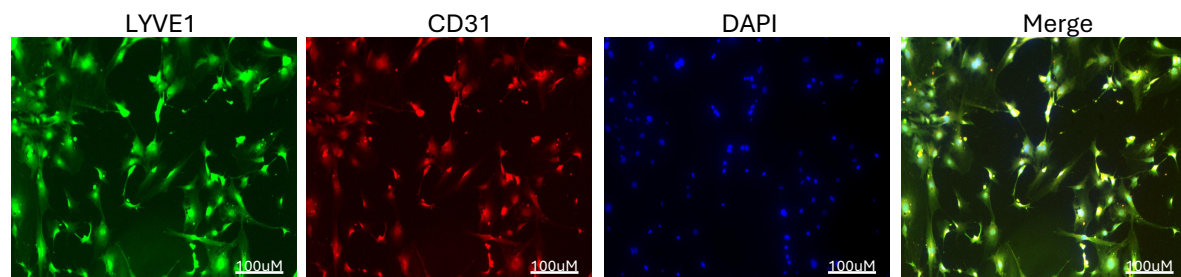

Supplement: S1 File — A. Immunohistochemistry showing double staining for LYVE-1 (marker of lymphatic endothelial cells) and CD31 (marker of endothelial cells) in isolated meningeal cell preparations. The majority of LYVE-1 ⁺ cells co-express CD31, indicating that the isolated LYVE-1 ⁺ population is predominantly composed of meningeal lymphatic endothelial cells. Representative images are shown. (PDF) [file pone.0342643.s001.pdf]

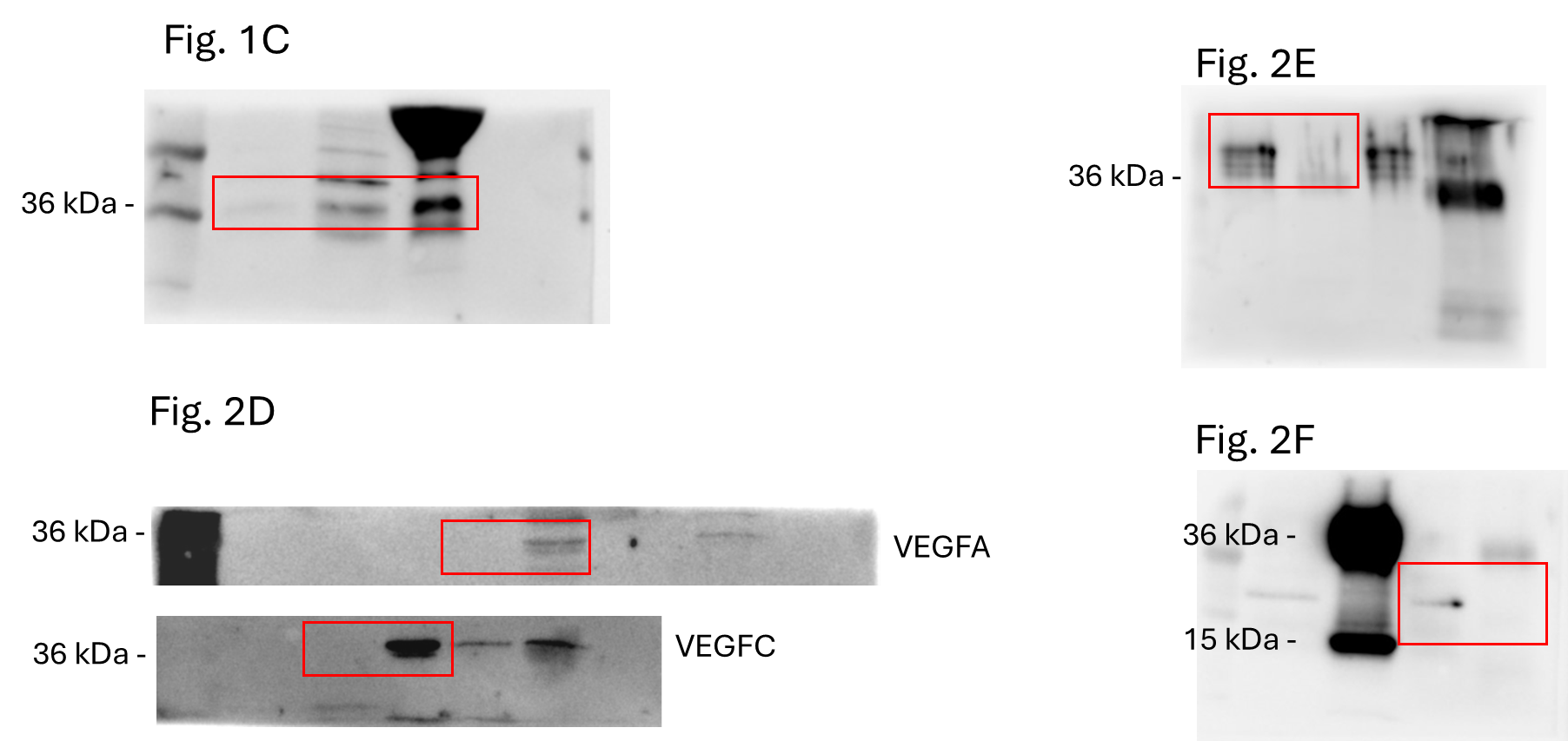

Supplement: S1 Fig — (PNG) [file pone.0342643.s002.png]
